# Demand analysis of telenursing among empty-nest elderly individuals with chronic diseases based on the Kano model

**DOI:** 10.3389/fpubh.2022.990295

**Published:** 2022-09-28

**Authors:** Yuan Yuan, Chunhua Tao, Ping Yu, Yanwei Wang, Akio Kitayama, En Takashi, Kiyoko Yanagihara, Jingyan Liang

**Affiliations:** ^1^School of Nursing & School of Public Health, Yangzhou University, Yangzhou, China; ^2^Nagano College of Nursing, Komagane, Japan; ^3^Affiliated Hospital of Yangzhou University, Yangzhou, China; ^4^Institute of Translational Medicine, Medical College, Yangzhou University, Yangzhou, China; ^5^Jiangsu Key Laboratory of Integrated Traditional Chinese and Western Medicine for Prevention and Treatment of Senile Diseases, Yangzhou University, Yangzhou, China

**Keywords:** telenursing, empty-nest, elderly, chronic diseases, Kano model

## Abstract

**Aim:**

The increase in empty-nest elderly individuals with chronic diseases poses a major challenge to the provision of public health services in China. Telenursing can effectively relieve the pressure of public health services to a certain extent. This study aims to explore the telenursing needs of empty-nest elderly individuals with chronic diseases based on the Kano model to provide references for improving the quality of telenursing.

**Methods:**

Participants were selected from five rural communities and five urban communities in Yangzhou and Nantong, Jiangsu Province, China. A total of 348 empty-nest elderly individuals with chronic diseases were included. The participants received a sociodemographic characteristics questionnaire, and their telenursing needs were surveyed and analyzed based on the Kano model.

**Results:**

Of the 15 quality attributes evaluated by the participants, 3 telenursing services were categorized as “must-be quality”, 5 were categorized as “one-dimensional quality”, 5 were categorized as “attractive quality”, and 2 were categorized as “indifferent quality”. The proportion of individuals who desired telenursing services ranged from 47.41 to 83.62%, the better values (satisfaction) ranged from 35.29–83.98%, and the worse values (dissatisfaction) ranged from 10.91 to 63.27%. There were no significant differences in any items of telenursing needs for between participants in Yangzhou and Nantong (all *P* > 0.05), and there were also no significant differences in all items between rural and urban communities (all *P* > 0.05).

**Conclusion:**

Based on the Kano model, it was found that empty-nest elderly individuals with chronic diseases had a positive attitude toward telenursing and that they had different levels of need for different telenursing services. These findings provided a theoretical basis for medical decision-makers to formulate medical policies and provided a scientific foundation for nursing managers to improve telenursing services to meet the needs of the empty-nest elderly individuals with chronic diseases.

## Introduction

The decrease in fertility rates coupled with the increase in life expectancy has led to large changes in the age structure of the population (1). At present, the increase in the number and proportion of the elderly population has become a global problem (2, 3). China is one of the world's fastest-aging countries (4). According to the seventh National Census in 2020, 264.02 million people (approximately 18.7% of the total population) are now aged 60 years or older, among them, the population aged 65 and above is 190.64 million, accounting for 13.5% of the total population (5). The increasing health needs of elderly individuals, coupled with the decreasing numbers of people with working age, have led to major challenges to public health services in China (6).

Population aging brings many challenges to societies and economies, one of the most significant challenges is the increase in the number and proportion of people with chronic diseases (7). In China, 150 million people (nearly 90% of the aging population) had a chronic disease by the end of 2018, according to China's National Health Commission (5). Another social and economic challenge associated with the aging population is the increasing number and proportion of empty nesters (8). The term “empty nester” refers to elderly individuals (age over 65 years of age) who live alone or live with their spouse because they do not have children or because their children are married or work outside for a long time (9). In China, it is estimated that the proportion of empty-nest elderly individuals will reach 90% by 2030, which means that almost all elderly families will be empty-nest families (10). Empty nesters face the uncertainty of financial support and spiritual consolation (11). Thus, paying attention to empty nesters is an essential component of public health.

Empty-nest elderly individuals with chronic diseases often suffer from poor health conditions and quality of life. In recent years, because of the rapid development of information technology and medical technology, the feasibility of telenursing has improved. Telenursing can effectively overcome time and space constraints, reduce the economic load and improve the health situation of elderly individuals with chronic diseases. Many countries gradually use telenursing to expand elderly care (12, 13), especially during the COVID-19 pandemic (14). Smith et al. reported that remote intervention is an ideal choice for managing infectious diseases, as it can effectively reduce the contact between people and slow down the spread of the virus. Especially for elderly individuals with a previous medical history, remote intervention can provide routine care and avoid exposure in crowded hospitals or medical clinics (15). In China, telenursing research started late, and before we fully implement telenursing, it is important to understand the needs of patients.

**Figure 1:**
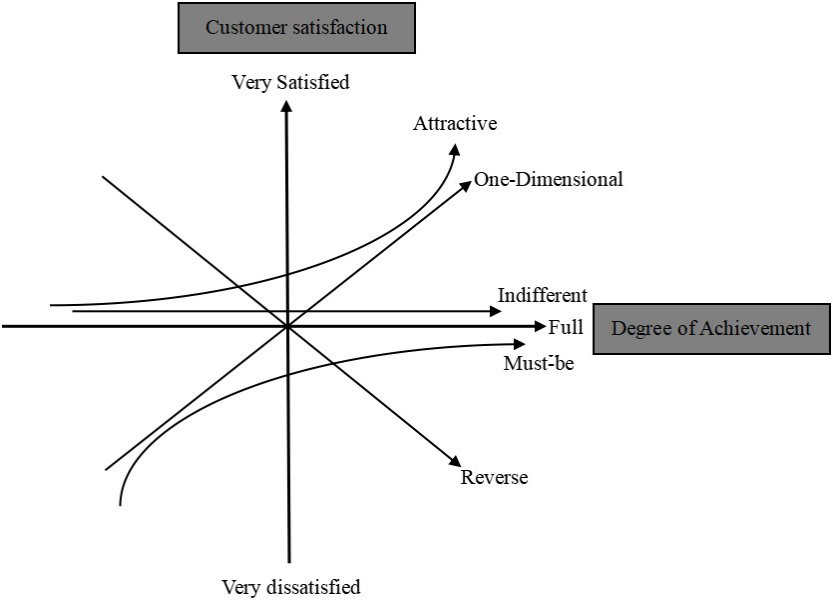
The Kano model.

The Kano model is an easy and effective method to recognize service attributes, and the model can accurately identify the service attributes of customers' demands (16). The Kano model was proposed by a Japanese professor named Kano in 1984 (17). In accordance with the relationship between the subjective feelings of customers and the objective performance of products, the Kano model divided the service attributes into must-be qualities (M), one-dimensional qualities (O), attractive qualities (A), indifferent qualities (I) and reverse qualities (R) (18), as shown in Figure 1. Must-be qualities are features that, if not provided, may make users dissatisfied, however, when they are provided, they will not improve users' satisfaction. One-dimensional qualities are features whose presence will improve users' satisfaction and whose absence will reduce users' satisfaction. Attractive qualities are features that, if not provided, will reduce users' satisfaction and, if provided, can greatly improve users' satisfaction. Indifferent qualities are those that have no impact on users' satisfaction. Reverse qualities mean that users' satisfaction will decrease if these features are provided, and users' satisfaction is inversely proportional to the degree of provision (19).

In recent years, researchers in the United States, China, Pakistan and many other countries have successively applied the Kano model to the medical industry to judge patients' demand for medical services (20–22). The purpose of this study is to explore the demands of telenursing for empty-nest elderly individuals with chronic diseases using the Kano model and to provide a theoretical basis for medical decision-makers to formulate medical policy and a scientific foundation for nursing managers to improve telenursing services to meet the needs of empty-nest elderly individuals with chronic diseases.

## Participants and methods

### Participants

From January 1st to March 31st, 2021, empty-nest elderly individuals with chronic diseases from different communities in Yangzhou and Nantong were selected. Participants were eligible if they met the following inclusion criteria: (1) age ≥ 65 years; (2) with at least one chronic disease; (3) living alone or with their spouses, not living with their children; (4) living in the investigated community for more than 12 months, that is, the permanent resident population of the community; and (5) participating in the survey voluntarily. Participants were excluded if they met any exclusion criteria: (1) in the acute stage of disease, or severe cardiopulmonary, renal insufficiency, or terminal disease stage; (2) with communication, cognitive, or mental disorders.

### Sample size calculation

According to the description of sample size calculation in Medical Statistics, the sample size of a cross-sectional study should be 10**–**20 times the number of independent variables. According to the results of previous studies, the number of independent variables in this study was determined to be 15. Assuming a loss to follow-up rate of no more than 20%, the sample size required for this study was (15^*^10)^*^1.2**–**(15^*^20)^*^1.2, that is, the sample size range was 180**–**360 cases, and 348 cases were finally included in this study.

### Survey questionnaire

The questionnaire in this study was divided into two parts. The first part was a self-designed sociodemographic characteristics questionnaire, including gender, age, education level, residence, living conditions and chronic diseases. The second part was a needs survey of telenursing for empty-nest elderly individuals with chronic diseases based on the Kano model, as shown in Table 1. It was self-designed based on the Kano model from Kano (23) and the “Salus vision” telenursing system from Kitayama (24). Through “Salus vision”, medical staff could implement remote education and training and monitoring and so on. The telenursing system has been used in many cities in China, the Philippines and Japan, and it has achieved satisfactory results. Fifteen pairs of questions were included in the second section, each pair of questions includes forward questions (Functional Form) and reverse questions (Dysfunctional Form), for example, “1.A. If you received remote education on home safety prevention (fall prevention, fraud prevention, etc.), how would you feel?” and “1.B. If not, how would you feel?”. For each question, the respondent could choose the most suitable answer from “I like it that way”, “It must be that way”, “I am neutral”, “I can live with it” and “I dislike it that way”. There were 25 possible results, and each result corresponded to a Kano attribute. “M” indicates must-be attributes, “O” indicates one-dimensional attributes, “A” indicates attractive attributes, “I” indicates indifferent attributes, “R” indicates reverse attributes, and “Q” indicates questionable answers, as shown in Table 2. The Cronbach's alpha value of the questionnaire was 0.84 and the 2-week test-retest reliability coefficient was 0.82. The questionnaire has been used in other studies and proved to have good reliability and validity (25).

**Table 1:** Kano questionnaire.

**Number**	**Attribute**
1	Remote education on home safety prevention (fall prevention, fraud prevention, etc.)
2	Teletraining on care skills (mastering physical and mental care skills to enhance self-care and family and community support)
3	Remote lectures about disease prevention
4	Remote screening for diseases (screening for diseases after the relevant indicators are entered into the database)
5	Distance intervention for disease risk factors
6	Remote monitoring of vital signs and sleep, etc.
7	Remote diagnosis of diseases
8	Remote health counseling
9	Remote calls for life needs (turning over, patting on the back, etc.)
10	Remote calls for nursing needs (pressure injury care, etc.)
11	Remote one-button emergency caller
12	Remote emergency assistance arrangement (Arranging emergency measures according to the condition and opening “green passages” with the corresponding hospitals)
13	Remote rehabilitation guidance
14	Remote return visits and related health education
15	Regular family visits (physical examination, etc.)

**Table 2:** Kano evaluation table.

**Functional form**	**Dysfunctional form**				
	**I like it that way**	**It must be that way**	**I am neutral**	**I can live with it**	**I dislike it that way**
I like it that way	Q	A	A	A	O
It must be that way	R	I	I	I	M
I am neutral	R	I	I	I	M
I can live with it	R	I	I	I	M
I dislike it that way	R	R	R	R	Q

### Investigation methods

After homogenization training, researchers began to conduct the investigation. They selected five rural communities and five urban communities in Nantong and Yangzhou, respectively. As a first step, researchers visited each community leader to understand the situations of the elderly individual's households in each community. However, community leaders did not fully know whether the elderly residents suffered from chronic diseases or whether they were empty nesters, and researchers had to include all older residents in subsequent steps. Then, the elderly residents' house numbers were written down on the paper by the researchers, and each house number corresponded to an Arabic number. Researchers used Excel to randomly select 30 Arabic numbers (house numbers) in each community. Among the 30 households, some were unwilling to accept the survey, some were not at home, and some did not meet the inclusion criteria. Ultimately, researchers could not obtain 30 questionnaires in each community. Researchers went to the community meeting sites or participants' homes and explained the aims and methods of this study to each participant and then instructed them to fill out the questionnaire after providing consent. After the completion of the questionnaire, the researcher conducted an interview with each participant about the questionnaire to further understand the responses. Finally, 352 of the 600 households met the inclusion criteria and were willing to participate in the survey. Therefore, in this study, 352 questionnaires were sent out. However, one participant's questionnaire was incomplete, and three participants provided questionable answers. Thus, 348 valid questionnaires were included, yielding an effective rate was 98.86%.

### Statistical analysis

Data were analyzed with IBM SPSS Statistics 26.0 software. Frequency and percentage were used to describe the sociodemographic characteristics of the participants. The telenursing needs attributes of participants were described based on the Kano model. The differences in telenursing demands between Yangzhou and Nantong, rural and urban communities were statistically analyzed by the rank sum test.

## Results

### The sociodemographic characteristics of the participants

**Figure 2:**
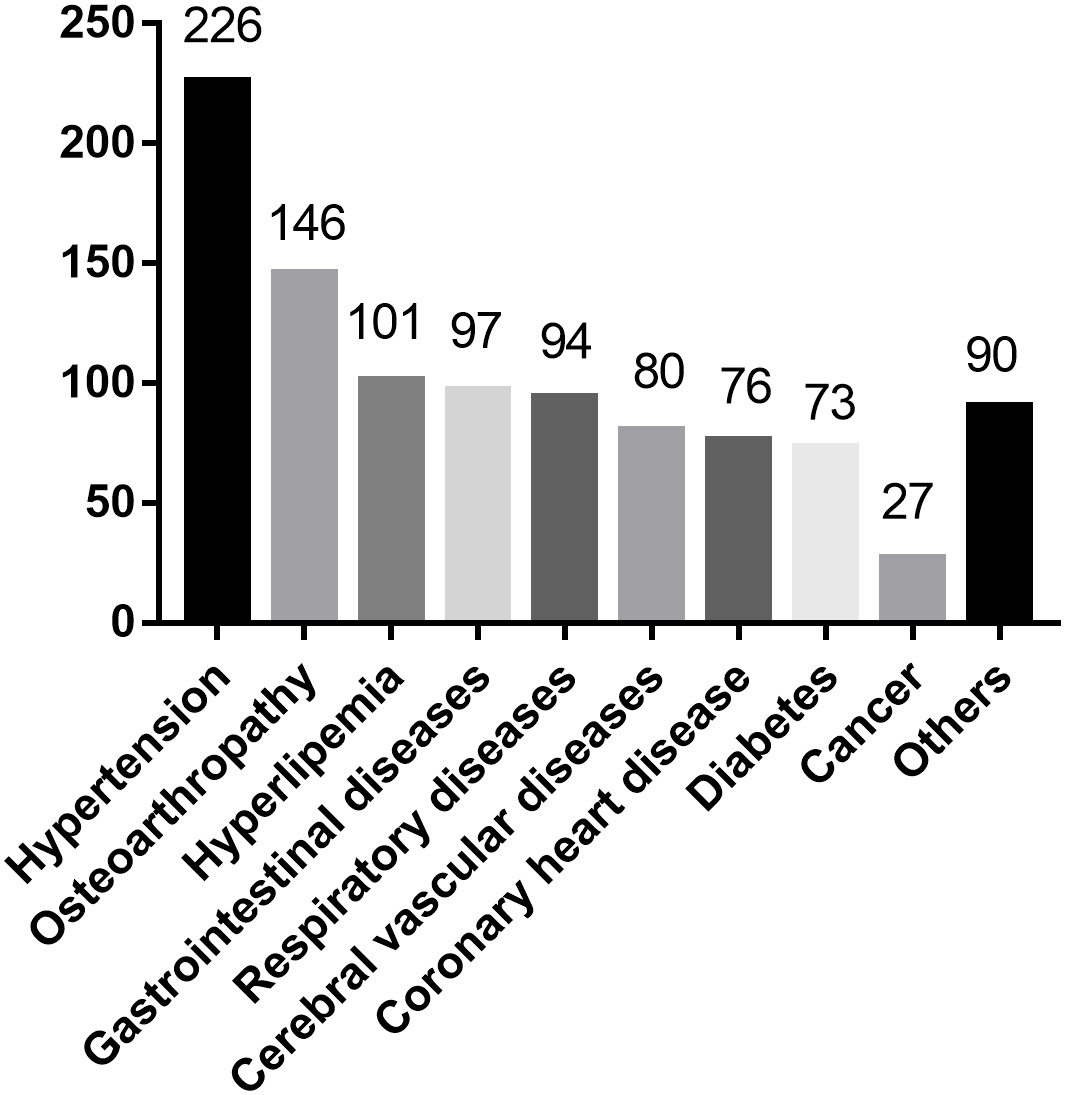
Distribution of participants' chronic disease types.

In total, 348 empty-nest elderly individuals with chronic diseases completed the questionnaires. Of all participants, females accounted for 56.32% of the participants. Their average age was 72.68 ± 6.00 years old: 221 participants were between 65 and 74 years old; 113 participants were between 75 and 84 years old; and 14 participants were over 85 years old. Among these participants, 78 persons were illiterate or barely literate; 97 persons received primary education; 92 persons received junior high school education; 44 persons received senior high school or technical secondary school education; and 37 persons had a junior college diploma or higher. Of all participants, 87 (25.00%) were from urban communities in Yangzhou; 88 (25.29%) were from urban communities in Nantong; 86 (24.71%) were from rural communities in Yangzhou; 87 (25.00%) were from rural communities in Nantong. A total of 155 people lived alone; and 193 people lived with their spouses. The distribution of participants' sociodemographic characteristics is shown in Table 3, and the distribution of their chronic diseases is shown in Figure 2.

**Table 3:** Distribution of participants' sociodemographic characteristics (*N* = 348).

**Characteristics**	**Observation** **(N)**	**Percentage** **(%)**
**Gender**		
Female	196	56.32
Male	152	43.68
**Age group (years)**		
65–74	221	63.51
75–84	113	32.47
85 and above	14	4.02
**Education level**		
Illiterate or barely literate	78	22.41
Primary school	97	27.87
Junior high school	92	26.44
Senior high school or technical secondary school	44	12.64
Junior college diploma or higher	37	10.63
**Residence**		
Urban residents (Yangzhou)	87	25.00
Urban residents (Nantong)	88	25.29
Rural residents (Yangzhou)	86	24.71
Rural residents (Nantong)	87	25.00
**Current living conditions**		
Living alone	155	44.54
With spouse	193	55.46

### Evaluation of the attributes of telenursing needs using the Kano model

As shown in Table 4, from the Kano final category, we can determine that 3 telenursing services were categorized as “must-be quality”, 5 were categorized as “one-dimensional quality”, 5 were categorized as “attractive quality”, and 2 were categorized as “indifferent quality”. The category strength was calculated as the percent difference between the highest category and next highest category, which was between 4.89 and 34.20%. The total strength was calculated as the total percentage of responses in the must-be, one-dimensional and attractive categories, which were between 47.41 and 83.62%. The better (coefficient of satisfaction) value was calculated as Better = (A+O)/(A+O+M+I), where A, O, M, and I are the number of participants in the attractive, one-dimensional, must-be, and indifferent categories, respectively. The worse (coefficient of dissatisfaction) value was calculated as Worse = (O+M)/(A+O+M+I). The better and worse values were all between 0 and 1, the closer the better value was to 1, the higher the satisfaction was when this service was provided, and the closer the worse value was to 1 indicated that providing such an attribute would only prevent dissatisfaction. Values closer to 0 indicated that the service had very little effect on satisfaction or dissatisfaction.

**Table 4:** Evaluation of the attributes of telenursing needs using Kano model.

**Attribute**	**Category totals**						**Final category**	**Category strength** **(%)**	**Total strength** **(%)**	**Better** **(%)**	**Worse** **(%)**
	**M**	**O**	**A**	**I**	**Q**	**R**					
1	8	34	170	122	7	7	A	13.79	60.92	61.08	12.57
2	4	33	174	128	6	3	A	13.22	60.63	61.06	10.91
3	4	36	170	117	8	13	A	15.23	60.34	63.00	12.23
4	11	37	165	126	5	4	A	11.21	61.21	59.59	14.16
5	11	36	169	121	6	5	A	13.79	62.07	60.83	13.95
6	105	88	73	73	7	2	M	4.89	76.44	47.49	56.93
7	6	166	82	81	4	9	O	24.14	72.99	74.03	51.34
8	7	193	84	59	4	1	O	31.32	81.61	80.76	58.31
9	5	40	120	172	5	6	I	14.94	47.41	47.48	13.35
10	5	39	123	170	8	3	I	13.51	47.99	48.07	13.06
11	167	48	72	53	5	3	M	27.30	82.47	35.29	63.24
12	153	64	63	63	4	1	M	25.57	80.46	37.03	63.27
13	6	201	82	48	4	7	O	34.20	83.05	83.98	61.42
14	7	194	88	54	3	2	O	30.46	83.05	82.22	58.60
15	7	196	88	49	4	4	O	31.03	83.62	83.53	59.71

### Attributes on the better-worse plot

**Figure 3:**
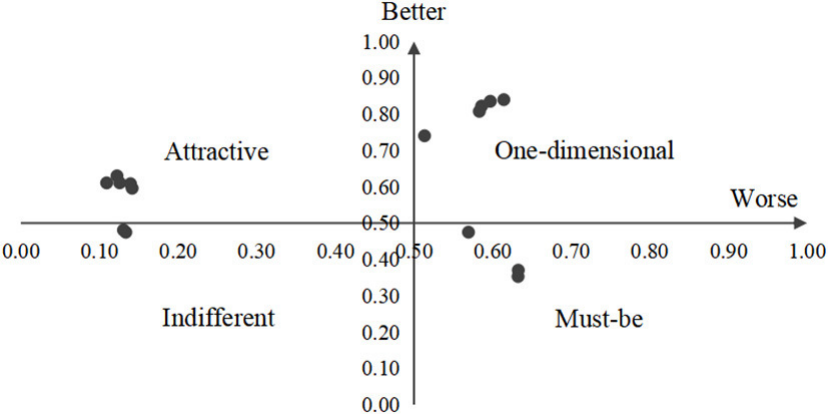
Attributes on the better-worse plot.

As seen from Figure 3, the service attributes were presented graphically based on the worse and better values on the x- and y-axes, respectively. Each attribute was represented as a point on the graph. The results of Better-Worse plot was consistent with the above findings.

### The differences in telenursing demands between Yangzhou and Nantong

As shown in Table 5, there were no significant differences in all items of telenursing needs for the participants in Yangzhou and Nantong (all *P* > 0.05).

**Table 5:** The differences in telenursing demands between Yangzhou and Nantong.

**Attribute**	**City**	**M**	**O**	**A**	**I**	**Q**	**R**	**Z**	**P**
1	Yangzhou	3	13	88	59	6	4	1.07	0.28
	Nantong	5	21	82	63	1	3		
2	Yangzhou	2	16	84	66	3	2	0.60	0.55
	Nantong	2	17	90	62	3	1		
3	Yangzhou	0	18	88	57	4	6	0.03	0.97
	Nantong	4	18	82	60	4	7		
4	Yangzhou	4	18	82	63	3	3	0.70	0.49
	Nantong	7	19	83	63	2	1		
5	Yangzhou	3	20	86	58	3	3	0.12	0.91
	Nantong	8	16	83	63	3	2		
6	Yangzhou	50	49	33	35	4	2	0.05	0.96
	Nantong	55	39	40	38	3	0		
7	Yangzhou	1	91	39	37	0	5	1.24	0.22
	Nantong	5	75	43	44	4	4		
8	Yangzhou	2	102	43	25	0	1	1.12	0.26
	Nantong	5	91	41	34	4	0		
9	Yangzhou	1	19	52	96	1	4	1.92	0.054
	Nantong	4	21	68	76	4	2		
10	Yangzhou	2	20	58	87	4	2	0.57	0.57
	Nantong	3	19	65	83	4	1		
11	Yangzhou	80	22	45	23	2	1	0.36	0.72
	Nantong	87	26	27	30	3	2		
12	Yangzhou	81	30	31	28	2	1	1.00	0.32
	Nantong	72	34	32	35	2	0		
13	Yangzhou	1	104	40	22	2	4	0.19	0.85
	Nantong	5	97	42	26	2	3		
14	Yangzhou	3	94	45	28	1	2	0.71	0.48
	Nantong	4	100	43	26	2	0		
15	Yangzhou	1	96	48	24	2	2	0.94	0.35
	Nantong	6	100	40	25	2	2		

### The differences in telenursing demands between rural and urban communities

As shown in Table 6, there were no significant differences in all items of telenursing needs for the participants in rural and urban communities (all *P* > 0.05).

**Table 6:** The differences in telenursing demands between rural and urban communities.

**Attribute**	**Community**	**M**	**O**	**A**	**I**	**Q**	**R**	**Z**	**P**
1	Rural	4	16	81	65	4	3	0.85	0.40
	Urban	4	18	89	57	3	4		
2	Rural	2	14	81	71	2	3	1.74	0.08
	Urban	2	19	93	57	4	0		
3	Rural	2	16	83	60	4	8	0.92	0.36
	Urban	2	20	87	57	4	5		
4	Rural	4	17	83	64	2	3	0.74	0.46
	Urban	7	20	82	62	3	1		
5	Rural	5	18	83	61	2	4	0.34	0.74
	Urban	6	18	86	60	4	1		
6	Rural	53	40	35	40	3	2	0.54	0.59
	Urban	52	48	38	33	4	0		
7	Rural	2	84	39	40	3	5	0.22	0.82
	Urban	4	82	43	41	1	4		
8	Rural	4	92	44	31	1	1	0.58	0.56
	Urban	3	101	40	28	3	0		
9	Rural	2	17	66	81	4	3	0.02	0.98
	Urban	3	23	54	91	1	3		
10	Rural	3	19	64	82	3	2	0.58	0.56
	Urban	2	20	59	88	5	1		
11	Rural	79	23	39	27	3	2	0.98	0.33
	Urban	88	25	33	26	2	1		
12	Rural	75	30	34	32	1	1	0.33	0.74
	Urban	78	34	29	31	3	0		
13	Rural	3	93	46	26	2	3	1.28	0.20
	Urban	3	108	36	22	2	4		
14	Rural	3	94	45	26	3	2	0.78	0.44
	Urban	4	100	43	28	0	0		
15	Rural	4	93	45	28	2	1	0.78	0.44
	Urban	3	103	43	21	2	3		

## Discussion

Elderly individuals often have a high prevalence of chronic diseases, especially empty-nest elders (26). Hypertension is a widespread and severe global public health issue (27). In this study, for the participants, the prevalence of hypertension was the highest, up to 64.94%. In fact, some respondents had hypertension but did not know it because they had no symptoms. Therefore, it was quite possible that the prevalence of hypertension in empty-nest elderly individuals with chronic diseases was higher than 64.94%. Hypertension is also a major independent, progressive hazard for many chronic non-communicable diseases, specifically for cardiovascular diseases, causing substantial health and economic losses globally, which needs more attention (28).

It was an unexpected finding that in this study, osteoarthropathy ranked second in the prevalence of all chronic diseases, which had not been seen in previous reports. Through qualitative interviews, we found that the main reason was that the chronic disease situations were filled in by the elderly individuals themselves. Many elderly people did not think they were suffering from hyperlipidaemia, diabetes and other diseases because they did not participate in the physical examination or did not understand the physical examination reports. However, many elderly people had physical pain, such as knee or shoulder pain, so they thought they had osteoarthropathy. We can learn two things through the results. First, we need to continuously improve the health literacy of elderly individuals to improve their awareness of the disease or prevent the high-risk factors for the disease in advance (29). Second, the problem of physical pain among elderly individuals is serious (30). Previous studies found that physical pain affects emotion and sleep, while emotion and sleep also react to the body, making people feel more pain, which then affects the overall health status (31). Therefore, physical pain is another problem worthy of our attention.

Cancer is the leading cause of death in China. The Global Burden of Diseases, Injuries, and Risk Factors Study 2019 (GBD 2019) reported that from 2010 to 2019, these represented a 26.3% increase in new cases and a 20.9% increase in deaths of cancer globally (32, 33). However, in this study, the prevalence of cancer was particularly low, only 7.76%. There were three main reasons. First, the detection rate of cancer in China was lower than that in developed countries, so many cancer patients did not know they had cancer in this study. Second, the family members of cancer patients would hide their condition from patients out of their protection. Third, some patients thought this was their privacy and would choose to hide it from researchers. We recommend enlarging the coverage of effective screening, educating, and vaccination programs in the future. Whether family members hid from patients or patients hid from researchers, to some extent, it was out of fear and avoidance of cancer. Therefore, in the future, we need to increase the popularization of cancer-related knowledge and improve the public's awareness of cancer, alleviate their fear and avoidance, and face treatment and nursing more actively, which may be more conducive to their health (34).

The results presented in Table 4 show that among the needs of telenursing services for empty-nest elderly individuals with chronic diseases, three aspects were categorized as “must-be qualities”, five aspects were categorized as “one-dimensional qualities”, five aspects were categorized as “attractive qualities”, two aspects were categorized as “indifferent qualities”, and no aspect was categorized as a “reverse quality”. This shhows the participants had a positive attitude toward telenursing services.

### Must-be qualities

“Must-be qualities” are the basic characteristics of services or products. Participants think they are services that are taken for granted or that must be possessed (17). In this study, “Remote monitoring of vital signs and sleep”, “Remote one-button emergency caller” and “Remote emergency assistance arrangement” were “must-be qualities”. This shows that participants had a very high demand for telenursing services in these three aspects. Compared with other telenursing services, these three items had a stronger emphasis on the monitoring of unexpected conditions or handling of emergencies of patients. Post-survey interviews revealed that most empty-nest elderly individuals with chronic diseases were anxious about the uncertainty of their health condition. Some participants or people they know had experienced sudden illnesses such as heart attacks and strokes, which made them fear unexpected situations. In this study, all the participants lived alone or just lived with their spouses, they expected eagerly, even taking it for granted, that medical personnel could provide monitoring anytime and anywhere. In addition, they were eager for timely and effective treatment when they suddenly got sick. Most of them thought that without such three aspects of telenursing services, in case of emergency, their survival rate would be greatly affected (35).

### One-dimensional qualities

The satisfaction of participants is directly proportional to “one-dimensional qualities”, that is, the more “one-dimensional qualities” that are provided, the more satisfied the participants are. In contrast, when the provision is insufficient, the dissatisfaction will increase (36). In this study, “Remote diagnosis of diseases”, “Remote health counseling”, “Remote rehabilitation guidance”, “Remote return visits and related health education” and “Regular family visits” were “one-dimensional qualities”. Compared with other telenursing services, these five items had a stronger emphasis on the diagnosis of disease and health guidance after diagnosis. Post-survey interviews revealed several reasons for this phenomenon. (a) Even people who did not pay much attention to health care at ordinary times would still be anxious or want to take some measures to change once they were diagnosed. Remote health counseling, remote return visits and regular family visits can make the diseases be diagnosed in time, and the participants can receive treatment and nursing as soon as possible. (b) They believed that if they can receive online health guidance on diseases, it is very convenient and will save more time. This also reduces the risk of infection, especially during the COVID-19 pandemic (37).

### Attractive qualities

“Attractive qualities” means that if they are provided, participants will feel very surprised and satisfied. If they are inadequate, users will not feel dissatisfied (38). In this study, “Remote education on home safety prevention”, “Tele-training on care skills”, “Remote lectures about disease prevention”, “Remote screening for diseases” and “Distance intervention for disease risk factors” were attractive qualities. Compared with other telenursing services, these five items had a stronger emphasis on the prevention of diseases. Post-survey interviews revealed that empty-nest elderly individuals with chronic diseases did not have a strong awareness of disease prevention, so they thought it would be good if the medical staff provided some disease prevention knowledge, but if the medical staff did not tell them, they would not be dissatisfied. Therefore, it also reminded us that in the process of providing telenursing in the future, we need to gradually change the inherent concepts of elderly individuals so that they can understand that, to a certain extent, disease prevention is more important than treatment, similar to how fire prevention is more important than firefighting. Once a fire occurs, it will cause certain losses; the same concept applies to the body, as once we get sick, it will cause certain damage.

### Indifferent qualities

“Indifferent qualities” are services that users do not care much about whether they are provided (39). “Remote calls for life needs” and “Remote calls for nursing needs” were indifferent qualities. Post-survey interviews revealed that there were several reasons for this phenomenon. (a) Although participants lived alone or just with their spouses, most of the elderly people thought that if they needed help, such as turning over, patting on the back, their children or grandchildren would come. They thought their family support system was relatively complete. If it was not for family members to do these things, it would make them feel a little embarrassed and make others feel that their children are not filial. (b) Most participants thought they did not have pressure injuries or other symptoms and signs for the time being, so they could live independently basically, or their spouse would help them, and they did not need others to help them. In fact, a previous study showed that the spouses of elderly individuals were often also old, and the help they could provide to their spouse was limited; telenursing can compensate for this (40).

### Attributes on the better-worse plot

All the services were divided into four quadrants. Similar to the evaluation of the attributes of telenursing needs using the Kano model, three aspects were categorized as “must-be qualities”, five aspects were categorized as “one-dimensional qualities”, five aspects were categorized as “attractive qualities”, two aspects were categorized as “indifferent qualities”, and no aspect was categorized as a “reverse quality”. The Better-Worst Plot gives a more intuitive feeling. As Kano (41) mentioned, service quality attributes have their own life cycle and change over time. “Attractive attributes” that surprised or amazed users initially might gradually become “one-dimensional attributes” and eventually become “must-be attributes”. This reminds us that the telenursing service for patients is not unchanging. We need to understand the needs of patients dynamically to provide more suitable telenursing services to be more conducive to the satisfaction and health of patients.

## Conclusion

Based on the Kano model, it was found that empty-nest elderly individuals with chronic diseases had a positive attitude toward telenursing and that they had different levels of need for different telenursing services. This study provided a theoretical basis for medical decision-makers to formulate medical policy and provided a scientific foundation for nursing managers to improve telenursing services to meet the needs of empty-nest elderly individuals with chronic diseases.

## Data availability statement

The original contributions presented in the study are included in the article/supplementary material, further inquiries can be directed to the corresponding author.

## Ethics statement

The studies involving human participants were reviewed and approved by Ethics Committee of School of Nursing, Yangzhou University (YZUHL2019001). The patients/participants provided their written informed consent to participate in this study.

## Author contributions

YY, CT, and JL designed the study protocol. YY, CT, and PY performed the data collection. AK, ET, and KY performed statistical analysis and interpretation. YY and YW drafted the manuscript. JL revised the article. All authors read and approved the final manuscript.

## Funding

This work was supported by the National 2018 local universities science, education, culture and health introduction project-high-end foreign experts project (GDW20183200386), Key R&D projects of Yangzhou (YZ2020097), Open project of Key Laboratory of zoonosis in Jiangsu Province (HX20014), and General program of natural science research in Colleges and universities of Jiangsu Province (21KJD320005).

## Conflict of interest

The authors declare that the research was conducted in the absence of any commercial or financial relationships that could be construed as a potential conflict of interest.

## Publisher's note

All claims expressed in this article are solely those of the authors and do not necessarily represent those of their affiliated organizations, or those of the publisher, the editors and the reviewers. Any product that may be evaluated in this article, or claim that may be made by its manufacturer, is not guaranteed or endorsed by the publisher.
